# Open-Source Tools for Neuromuscular Electrical Stimulation in Mouse Models: A Methodological Validation Study

**DOI:** 10.3390/muscles5020032

**Published:** 2026-04-30

**Authors:** Bana H. Odeh, Amanda L. Wellman, Michael Ameye, Zachary Atwood, Luke Gray, Aiswarya Saravanan, Havish Poluru, Morium Begam, Takako I. Jones, Renuka Roche, Joseph A. Roche

**Affiliations:** 1 Physical Therapy Program, Department of Health Care Sciences, Eugene Applebaum College of Pharmacy and Health Sciences, Wayne State University, Detroit, MI 48201, USAaiswarya.saravanan07@gmail.com (A.S.);; 2 Department of Pharmacology, School of Medicine, University of Nevada, Reno, NV 89557, USA; takakojones@med.unr.edu; 3 Occupational Therapy Program, School of Health Sciences, Eastern Michigan University, Ypsilanti, MI 48197, USA; rroche@emich.edu

**Keywords:** neuromuscular electrical stimulation, electrodes, stimulators, open-source, mouse models, dysferlinopathy

## Abstract

Neuromuscular electrical stimulation (NMES) is integral to studying muscle function in healthy and dystrophic mice. Certain commercial electrodes and laboratory stimulators used for NMES in mice are no longer in production. We developed and/or tested low-cost, open-source alternatives to discontinued commercial standards. We performed two studies—a comparison of electrodes and a comparison of stimulators. In the electrode study, in vivo NMES was applied to the left hindlimb ankle dorsiflexors in healthy C57BL/6J and dysferlin-null BLAJ mice using three electrode types: a previously available commercial electrode, a custom 3D-Printed electrode, and a custom Pen electrode assembled from off-the-shelf components. Twitch and tetanic torque were measured and compared using two-way repeated-measures ANOVA. Twitch torque differed by electrode type (*p* = 0.031), with lower values observed for the Pen electrode compared with the 3D-Printed electrode (e.g., 573 ± 72 vs. 666 ± 70 mN.mm in C57BL/6J mice), whereas tetanic torque did not differ significantly between electrode types (*p* = 0.060). In the stimulator study, twitch and tetanic contractions were elicited using the open-source StimJim stimulator and compared with contractions elicited by the discontinued Grass S48 stimulator. Twitch torque was lower with the StimJim (588 ± 107 mN.mm) compared with the Grass S48 (698 ± 116 mN.mm; *p* < 0.001), whereas tetanic torque values were not statistically different (*p* = 0.055). These findings indicate that open-source electrodes and stimulators can produce similar maximal tetanic torque under the tested conditions, although differences in twitch torque and stimulation parameters should be considered. These results reflect a methodological validation of accessible tools rather than a formal equivalence analysis.

## 1. Introduction

Neuromuscular electrical stimulation (NMES) is widely used in preclinical studies of muscle function, particularly in rodent models [1,2,3,4,5,6]. In mice, NMES protocols are frequently applied to the ankle dorsiflexor muscle group to evaluate contractile function under baseline and post-injury conditions [4,7]. Reproducible and maximal NMES requires standardized electrodes as well as a laboratory stimulator capable of delivering properly conditioned electrical pulses.

**Figure 1:**
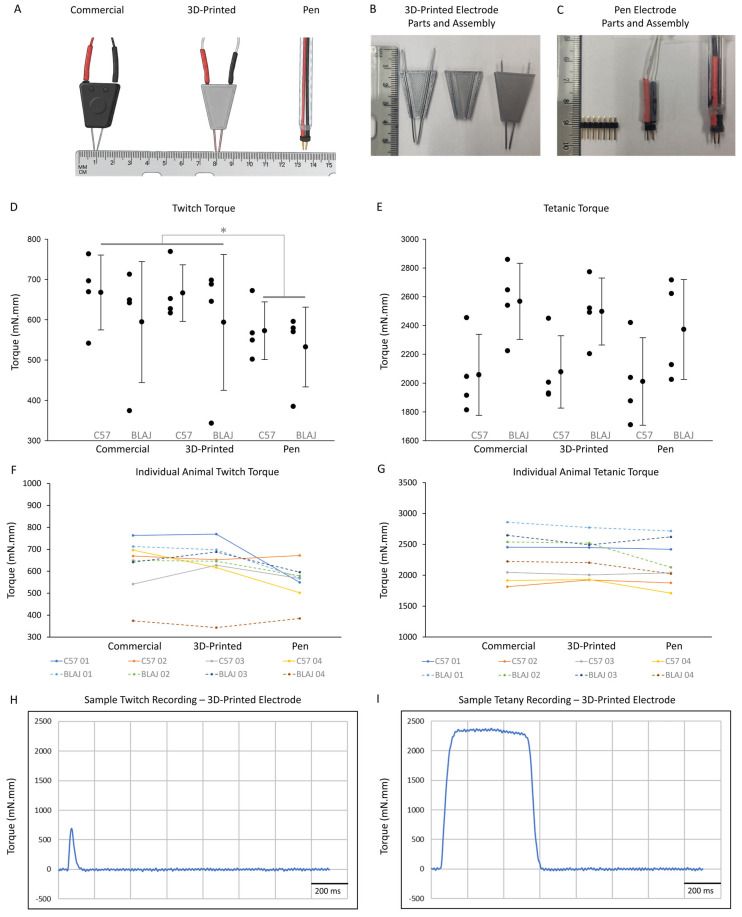
Design, fabrication, and functional testing of custom electrodes for neuromuscular electrical stimulation (NMES) in mice. (**A**) Side-by-side comparison of the three electrode designs evaluated in this study (Commercial, 3D-Printed, and Pen). Created in BioRender. Roche, J. (2026) https://BioRender.com/jik0cbt. (**B**,**C**) Assembly details and component views of the 3D-Printed and Pen electrodes. (**D**,**E**) Twitch and tetanic torque generated during NMES of the left hindlimb ankle dorsiflexors in adult C57BL/6J and BLAJ mice using the three electrode types. Individual data points represent single animals (*N* = 4 per strain) and bars denote mean ± SD. A significant main effect for electrode type was observed for twitch torque (two-way repeated-measures ANOVA; *, *p* = 0.031 for the pen electrode compared to the other two electrodes), with lower twitch torque produced using the Pen electrode. No significant main effect for electrode type was observed for tetanic torque, and no interaction effect was observed between electrode and mouse strain. (**F**,**G**) Individual animal twitch and tetanic torque values across electrode conditions. (**H**,**I**) Representative twitch and tetanic torque traces obtained using the 3D-Printed electrode.

A previously available bipolar electrode (Simple Electrode, BS4 50–6824, Harvard Apparatus; labeled as “Commercial” in our data sets) has been used for NMES of the hindlimb dorsiflexors in mice (Figure 1A, left) [5], and has been studied here as a reference comparator. Recent correspondence with the manufacturer confirmed that this product is no longer in production. This discontinuation creates challenges for consistency and reproducibility in NMES-based studies. To address this issue, we developed and tested two custom-built alternatives (Figure 1A). The first is a 3D-Printed electrode, which has stainless steel wire terminals enclosed within a rigid printed housing (Figure 1A, middle). The second is a Pen electrode assembled from male jumper wire headers from a commercial electronics kit (Figure 1A, right). Both designs were developed with reproducibility, accessibility, cost-effectiveness, simplicity of assembly, and ease of use in mind.

A laboratory stimulator previously used for NMES in mice, the Grass S48 (Grass Instruments, West Warwick, RI, USA), has also been discontinued. We therefore evaluated a low-cost, open-source electrical stimulator (StimJim) as a potential alternative [8]. The StimJim can be assembled by the investigator or purchased pre-assembled (e.g., from https://www.labmaker.org/collections/neuroscience/products/stimjimmaker (accessed on 1 April 2026) or https://open-ephys.org/stimulation/oeps-7520 (https://www.labmaker.org/collections/neuroscience/products/stimjimmaker (accessed on 1 April 2026)) at a substantially lower cost than traditional laboratory stimulators. Technical details, firmware, and Arduino-based control programs for the StimJim are publicly available through open-source resources [8]. We adapted this platform for NMES of the mouse ankle dorsiflexors and compared its performance with the Grass S48. The StimJim’s hardware and software and basic functionality have been described by its inventors in a preprint [8], but to the best of our knowledge, the use of this stimulator for NMES has not been described before. Therefore, the details that we provide in this paper on the StimJim may be useful to a wide audience.

In this paper, we present two studies. In the first (electrode study; partially published in a preprint), we compared the discontinued Commercial electrode with the 3D-Printed and Pen electrodes in age- and sex-matched C57BL/6J and dysferlin-null BLAJ mice (a mouse model of limb-girdle muscular dystrophy type 2B or R2) [9,10,11]. The objective of the electrode study was to evaluate the performance of custom-built electrodes relative to a discontinued commercial electrode under standardized experimental conditions. In the second (stimulator study), we compared the discontinued Grass S48 stimulator with the StimJim using C57BL/6J mice. The objective of the stimulator study was to evaluate the performance of the open-source StimJim relative to the Grass S48 within a defined NMES workflow. These studies were designed as methodological validation experiments focused on reproducibility and workflow feasibility rather than formal hypothesis testing, equivalence analysis, or engineering benchmarking. Accordingly, the findings should be interpreted within the context of methodological validation in a physiological system with inherent biological variability.

Despite the widespread use of NMES in murine muscle physiology, few studies have provided detailed validation of alternative electrode designs or stimulation platforms following discontinuation of legacy equipment. As a result, laboratories are often required to adapt workflows without clear guidance on performance comparability or reproducibility.

Our findings suggest that the open-source, low-cost electrode and stimulator options, which we tested, produced similar results to discontinued commercial comparators used in this study, in relation to eliciting peak muscle contractile force.

## 2. Results

**Figure 2:**
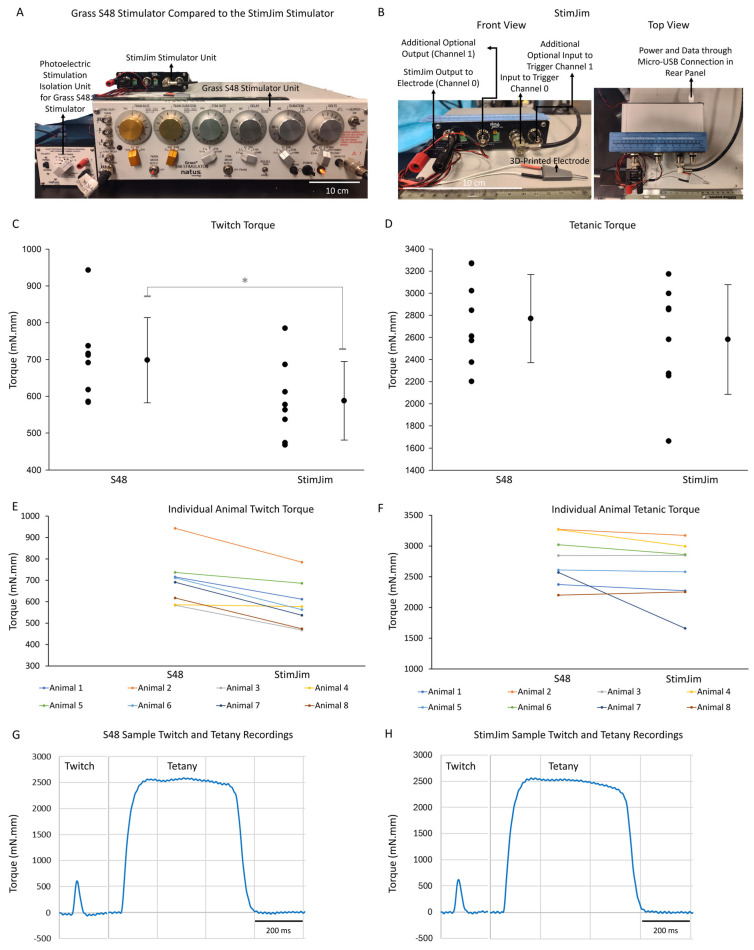
Functional comparison of the Grass S48 and StimJim stimulators for neuromuscular electrical stimulation (NMES) in mice. (**A**,**B**) Hardware used for NMES experiments, including the Grass S48 stimulator with stimulation isolation unit and the open-source StimJim stimulator. Despite its smaller size compared to the S48, the StimJim incorporates built-in stimulation isolation and provides two independent outputs for stimulation with corresponding inputs for timed triggering of electrical stimulation (**B**). The StimJim is powered and controlled via a micro-USB connection to a computer, and stimulation parameters are adjusted through an Arduino-based program rather than by manual dials (**B**). (**C**,**D**) Twitch and tetanic torque generated during NMES of the left hindlimb ankle dorsiflexors in adult mice using the Grass S48 or StimJim. Individual data points represent single animals and bars denote mean ± SD (*N* = 8). Twitch torque differed between stimulators (*, paired *t*-test, *p* < 0.001), whereas tetanic torque did not differ significantly. (**E**,**F**) Individual animal peak twitch and tetanic torque values across stimulator conditions. (**G**,**H**) Representative twitch and tetanic torque traces elicited by Grass S48 and StimJim during NMES.

Our results are presented in two parts—the electrode study (Figure 1) and the stimulator study (Figure 2). Results are presented to describe performance characteristics of each device within our experimental framework rather than to establish formal equivalence.

### 2.1. Electrode Study

In this study, we compared a commercial electrode with our 3D-printed and Pen electrodes.

#### 2.1.1. Twitch Torque for Electrode Study

Peak twitch torque values by strain (healthy C57BL/6J and dystrophic BLAJ) and electrode type (Commercial, 3D-Printed, and Pen; Figure 1A–C) are summarized in Figure 1D,F. Across both strains, twitch torque measurements were broadly similar between the Commercial and 3D-Printed electrodes, whereas the Pen electrode tended to produce lower values.

For C57BL/6J mice, mean ± SD peak twitch torque values were 668 ± 93 mN.mm (95% CI: 520 to 816) with the Commercial electrode, 666 ± 70 mN.mm (95% CI: 555 to 778) with the 3D-Printed electrode, and 573 ± 72 mN.mm (95% CI: 458 to 687) with the Pen electrode (Figure 1D).

For BLAJ mice, mean ± SD peak twitch torque values were 594 ± 150 mN.mm (95% CI: 356 to 833) with the Commercial electrode, 594 ± 168 mN.mm (95% CI: 356 to 832) with the 3D-Printed electrode, and 532 ± 99 mN.mm (95% CI: 375 to 690) with the Pen electrode (Figure 1D).

A two-way repeated-measures ANOVA revealed a significant main effect for electrode type (F(2,12) = 4.710, *p* = 0.031). Holm–Sidak post hoc testing indicated significantly lower twitch torque with the Pen electrode compared with the 3D-Printed electrode (Figure 1D). Twitch torque did not differ significantly between the Commercial and 3D-Printed electrodes.

There was no significant main effect for genotype (F(1,6) = 0.702, *p* = 0.434), indicating that overall twitch torque magnitude did not differ between C57BL/6J and BLAJ mice under the tested conditions. There was also no significant interaction between genotype and electrode (F(2,12) = 0.206, *p* = 0.817), suggesting that the relative performance of the electrode types was consistent across strains.

Inspection of individual animal data showed some inter-animal variability for the different types of electrodes, in both C57BL/6J and BLAJ mice, with the majority of animals showing a low twitch torque with the Pen electrode (Figure 1F). A representative twitch torque trace elicited with the 3D-Printed electrode is shown in Figure 1H.

In both C57BL/6J and BLAJ mice, for twitch torque, mean paired differences between the Commercial and 3D-Printed electrodes were negligible (~1 mN.mm in each strain; Cohen’s dz ≈ 0.02). Mean paired differences between the Commercial and Pen electrodes were larger (95 mN.mm in C57BL/6J mice; Cohen’s dz ≈ 0.75; 62 mN.mm in BLAJ mice; Cohen’s dz ≈ 0.98) (Supplementary Table S1).

#### 2.1.2. Tetanic Torque for Electrode Study

Peak tetanic torque values by strain and electrode type are summarized in Figure 1E,G. In contrast to twitch responses, tetanic torque values were similar across all three electrode types.

For C57BL/6J mice, mean ± SD tetanic torque values were 2057 ± 281 mN.mm (95% CI: 1610 to 2504) with the Commercial electrode, 2077 ± 251 mN.mm (95% CI: 1678 to 2477) with the 3D-Printed electrode, and 2011 ± 304 mN.mm (95% CI: 1528 to 2495) with the Pen electrode (Figure 1E).

For BLAJ mice, mean ± SD tetanic torque values were 2567 ± 265 mN.mm (95% CI: 2146 to 2989) with the Commercial electrode, 2497 ± 233 mN.mm (95% CI: 2126 to 2867) with the 3D-Printed electrode, and 2373 ± 347 mN.mm (95% CI: 1821 to 2924) with the Pen electrode (Figure 1E).

A two-way repeated-measures ANOVA showed no significant main effect for electrode type (F(2,12) = 3.590, *p* = 0.060). There was also no significant main effect for genotype (F(1,6) = 5.017, *p* = 0.066). There was no significant interaction between genotype and electrode (F(2,12) = 1.258, *p* = 0.319), indicating that electrode performance was not statistically different between the C57BL/6J and BLAJ strains.

Individual animal responses demonstrated consistent tetanic torque across electrode types (Figure 1G). A representative tetanic torque trace elicited with the 3D-Printed electrode is shown in Figure 1H

In both C57BL/6J and BLAJ mice, mean paired differences between the Commercial and 3D-Printed electrodes were small (−20 mN.mm in C57BL/6J mice; Cohen’s dz ≈ −0.32; 70 mN.mm in BLAJ mice; Cohen’s dz ≈ 1.08). Mean paired differences between the Commercial and Pen electrodes were 46 mN.mm in C57BL/6J mice (Cohen’s dz ≈ 0.40) and 195 mN.mm in BLAJ mice (Cohen’s dz ≈ 1.20) (Supplementary Table S1).

#### 2.1.3. Summary of Electrode Study Results

Collectively, these findings indicate that all electrode designs were capable of eliciting similar maximal tetanic torque measurements regardless of mouse strain under our specific experimental conditions.

### 2.2. Stimulator Study

In this study, we compared the Grass S48 and StimJim stimulators (Figure 2A,B).

#### 2.2.1. Twitch Torque for Stimulator Study

Peak twitch torque values obtained with the Grass S48 and StimJim stimulators are shown in Figure 2C,E. Across animals, twitch torque was consistently higher when stimulation was delivered using the Grass S48.

Mean ± SD twitch torque was 698 ± 116 mN.mm (95% CI: 601 to 795) for the Grass S48 and 588 ± 107 mN.mm (95% CI: 498 to 677) for the StimJim (Figure 2C).

A paired *t*-test demonstrated significantly greater twitch torque with the Grass S48 compared with the StimJim (*p* < 0.001, Figure 2C). Since measurements were paired within animals, this result indicates a difference between stimulators rather than inter-animal variability.

Individual animal responses are presented in Figure 2E. Most animals showed higher twitch torque with the Grass S48, although the magnitude of difference varied.

Representative twitch traces elicited with the Grass S48 and StimJim are shown in Figure 2G and Figure 2H, respectively. The twitch trace morphology was similar for both stimulators.

Mean paired differences between the Grass S48 and StimJim were 111 mN.mm, with higher twitch torque observed with the Grass S48 across animals. The corresponding within-animal effect size was large (Cohen’s dz ≈ 2.03) (Supplementary Table S2).

#### 2.2.2. Tetanic Torque for Stimulator Study

Peak tetanic torques obtained with the Grass S48 and StimJim stimulators are shown in Figure 2D,F. In contrast to twitch responses, tetanic torque values were closely matched between stimulators.

Mean ± SD tetanic torque was 2770 ± 398 mN.mm (95% CI: 2437 to 3103) for the Grass S48 and 2581 ± 496 mN.mm (95% CI: 2167 to 2996) for the StimJim (Figure 2D).

Because normality assumptions were not met, paired comparisons were performed using a Wilcoxon signed-rank test. Median tetanic torque was 2728 mN.mm for the Grass S48 and 2716 mN.mm for the StimJim. Peak tetanic torque elicited by the two stimulators did not reach statistical significance under tested conditions (*p* = 0.055), although the study may be underpowered to detect small but physiologically relevant differences.

Individual animal responses are shown in Figure 2F. Some animals exhibited slightly higher tetanic torque with one stimulator or the other, but no consistent directional bias was evident.

Representative tetanic traces elicited with the Grass S48 and StimJim are shown in Figure 2G and Figure 2H, respectively. The tetanic trace morphology was similar for both stimulators.

Mean paired differences between the Grass S48 and StimJim were 189 mN.mm, with variability across animals. The corresponding within-animal effect size was moderate (Cohen’s dz ≈ 0.61) (Supplementary Table S2).

#### 2.2.3. Summary of Stimulator Study Results

Taken together, these data indicate that while the Grass S48 produced higher twitch torque under the tested parameters, both stimulators elicited similar maximal tetanic contractions within the presented experimental conditions.

## 3. Discussion

### 3.1. Practical Implications

The primary goal of this work was to establish practical, reproducible approaches for murine NMES using accessible hardware rather than to perform a comprehensive engineering validation or equivalence analysis.

The purpose of this work was to address a practical and increasingly common barrier in murine neuromuscular physiology research. Several mission-critical NMES components, including a commercial bipolar transcutaneous electrode and the Grass S48 electrical stimulator, have been discontinued. This has created challenges for laboratories to continue or build on established and reliable workflows. To address this issue, we performed two complementary validation studies. First, we compared two low-cost custom bipolar electrodes (a 3D-Printed design and a Pen design) with a discontinued commercial electrode in healthy C57BL/6J mice and dysferlin-null BLAJ mice. Second, we compared the discontinued Grass S48 stimulator with the open-source StimJim and defined a practical workflow to achieve optimal stimulation and stable torque readings in mice.

Murine NMES is widely used in studies that quantify muscle function at baseline, after exercise/injury/perturbations, and in response to various therapeutic interventions. NMES-based dynamometry has supported work on studying the responses of healthy and dystrophic mice to rehabilitative [5,12], cell-based [13], genetic [14], and pharmacological interventions [15]. When essential equipment that has been used before for laboratory workflows becomes unavailable, laboratories are forced to find alternatives. Such alternatives must be systematically compared and vetted for comparison to past studies and to become the new standards in future studies. Furthermore, over the last decade, the cost of equipment for NMES in mice has increased to the extent that funding challenges can exclude researchers from pursuing promising muscle research projects. The present study evaluates alternatives that are inexpensive, open-source, and described in sufficient detail to permit replication by laboratories with limited NMES experience.

The electrode designs and stimulation workflow were developed to be reproducible, shareable, and less dependent on proprietary supply chains. The 3D-Printed electrode can be fabricated using standard 3D printers and materials, and the design files have been made open-source such that they can be shared and modified. The Pen electrode uses readily available and standard electronic kit components and can be assembled with minimal tools. The StimJim platform extends this approach to stimulation hardware by providing a lower-cost, open-source alternative to traditional laboratory stimulators.

These features are relevant for laboratories with limited financial and technical resources. In many settings, the cost of specialized stimulators and proprietary electrodes can be prohibitive, particularly when legacy devices are no longer manufactured. Lower-cost alternatives may allow more groups to perform quantitative muscle function studies. Open-source designs also permit inspection of geometry and materials. Because NMES outcomes depend on electrode–skin interface properties, spacing, and placement stability, this transparency can reduce uncertainty associated with knowledge transfer between laboratories.

### 3.2. Electrode Performance

Both custom electrodes produced tetanic torque values similar to the discontinued commercial electrode in both mouse strains under the tested conditions. Tetanic torque is a common functional endpoint because it reflects near-maximal recruitment and is relatively tolerant of small variations in placement once optimal stimulation and stable torque are achieved. In the present study, there was no significant main effect for electrode type for tetanic torque and no genotype-interaction. This supports the use of either custom electrode when maximal tetanic force is the primary outcome.

Twitch torque showed a different pattern. A significant main effect of electrode type was observed, with lower twitch values for the Pen electrode compared with the 3D-Printed electrode. Twitch measurements are more sensitive to current density, impedance, and small differences in contact geometry. The fixed spacing and rigid housing of the 3D-Printed electrode likely reduce placement variability, whereas the Pen electrode may allow subtle variation in terminal contact. From a practical standpoint, the 3D-Printed electrode is the more conservative option for studies emphasizing twitch measurements. The Pen electrode remains usable when tetanic torque is the primary endpoint or when rapid, low-cost fabrication is needed.

The absence of genotype effects or genotype–electrode interactions is also relevant. Dysferlin-deficient muscle can differ in susceptibility to contraction-induced damage, yet electrode performance did not appear to depend on genotype under the conditions tested. This suggests that the findings may extend across at least some disease models, although broader validation would be helpful.

### 3.3. Stimulator Performance and Workflow Considerations

For tetanic torque, the StimJim performed similarly to the Grass S48. Although mean values differed numerically, paired nonparametric analysis did not detect a significant difference and median values were nearly identical. This is an important observation because tetanic measurements are used to assess maximum contractile force production in many studies, and this is commonly used as a functional outcome.

Twitch torque was lower with the StimJim. Laboratories that rely on twitch amplitude or twitch-based indices may therefore need additional optimization when using this device. The difference likely reflects both hardware characteristics and workflow factors. The Grass S48 allows rapid adjustment using familiar dial/switch/knob-based controls, whereas the StimJim requires parameter entry through a serial interface. This introduces a learning curve, slows parameter exploration, and can be more time consuming than using dials and buttons to change stimulator settings. The StimJim’s inventors have shared an open-source, Python-based graphic user interface (GUI) in their file repository. In our hands, getting this (GUI) to work posed many challenges due to which we abandoned it. Laboratories that have expertise in application (“app”) and GUI design might be able to integrate the Arduino code that we have provided into an app or GUI, such that parameter optimization is made easier.

Electrode placement also influenced performance. With the StimJim, direct placement over the TA muscle belly produced more consistent recruitment than placement near the common peroneal nerve. This differs from our workflow with the Grass S48, where nerve-targeted electrode placement is always more effective than placement on the muscle belly. We do not fully understand the reason for this difference in behavior between the Grass S48 and the StimJim, but we speculate that it may relate to voltage compliance, impedance sensitivity, and waveform delivery under varying load conditions—e.g., the StimJim’s maximum voltage output through a single channel is 13–15 V [8]—with the Grass S48, we typically set the dial on the stimulator to output a range of 18–25 V, with stimulation isolation unit limiting the current regardless of resistance to 1–15 mA (Figure 2A). In addition to electrode placement on the TA muscle belly, increasing the pulse width on the StimJim from 0.1 ms to 1 ms improved stability in our workflow. This should not be interpreted as a universal requirement, but it provides a reproducible starting point. During our initial test runs of the StimJim, we observed an unexpected phenomenon of the torque output precipitously and abruptly dropping with nerve-targeted electrode placement. The torque drop seemed to happen more often when the StimJim was used in the constant voltage mode versus constant current mode (done through Arduino code; Constant Voltage and Constant Current Pulser, respectively; Supplementary Materials). Therefore, we chose to use the constant current mode as our standard.

A key methodological distinction in the stimulator study is that pulse duration differed between devices, with the Grass S48 used at 0.1 ms and the StimJim used at 1 ms. This difference arose from pilot testing in which longer pulse duration improved stability and reproducibility of torque measurements with the StimJim. Accordingly, the comparison should be interpreted as a workflow-optimized methodological comparison rather than a strict parameter-matched or engineering comparison. Differences observed in twitch torque may therefore reflect pulse duration and effective stimulus delivery in addition to device-specific characteristics.

### 3.4. Relevance to Prior Methods

Murine NMES underlies a range of established training and injury paradigms, including concentric and eccentric protocols and medium-strain forced eccentric exercise models. It is also central to dosage-adjusted resistance training approaches aimed at studying muscle adaptation. In these contexts, reliable stimulation is a prerequisite for meaningful interpretation of torque outcomes.

Prior work has highlighted the difficulty of consistently locating the common peroneal branch of the sciatic nerve using transcutaneous electrodes, particularly for new users [5]. The fact that the StimJim elicits similar maximum tetanic torque with muscle belly electrode placement compared to the Grass S48 could be leveraged by users who might not have the skill to perform nerve-targeted placement. It must be noted here that when motor innervation to a muscle is intact, motor axons will be excited before muscle fibers even when electrodes are placed directly on the muscle belly since motor axons are more excitable than muscle fibers [16]. The developers of the StimJim have indicated that it might be possible to connect the two output channels of the StimJim to increase voltage output—this is worth exploring in future studies to ascertain if twitch stimulation can be brought closer to the Grass S48.

### 3.5. Study Limitations

The limitations of this project include modest sample sizes, assessment of only one muscle group, and the absence of a dysferlinopathic mouse group for the stimulator study. Nonetheless, our work demonstrates that open-source materials, such as our 3D-Printed and Pen electrodes, as well as the StimJim, can be suitable replacement for prior standards, at least for assessing maximal NMES-elicited contractile force production. The stimulator comparison reflects a specific workflow and parameter set rather than a comprehensive characterization of device capability. Laboratories using different electrode geometries, lead lengths, or isolation strategies may observe different results. The studies were not blinded because device differences were obvious. Although torque measurement is objective, operator decisions could still introduce minor biases. We did not perform inter-laboratory reliability testing—such testing would strengthen external validity. Only male mice were studied—while this aligns with much prior literature, it misses out on addressing sex as a biological variable, which will be prioritized in follow-up studies. We did not systematically measure electrode–skin impedance, skin temperature, or small positioning differences, all of which can influence NMES outcomes. The electrode and stimulator studies were conducted in separate cohorts that differed in age. Although comparisons were performed within animals in each study, age-related differences in muscle excitability and contractile properties may influence the generalizability of findings across experimental contexts. In the stimulator study, since we did not study young and dystrophic cohorts as we did for the electrode study, the stimulator study findings may not extrapolate fully to young and dystrophic animals. Finally, the stimulator comparison incorporated different pulse durations between devices, which limits interpretation as a direct hardware-only comparison.

The electrodes and stimulators evaluated in this study were developed and tested for preclinical use, and the present findings are therefore limited to mouse models. Additional testing and optimization will be required to determine applicability across other species and experimental contexts.

Since standardized methodological descriptions of murine NMES workflows are limited, much of the available procedural detail is derived from a relatively small number of detailed reports, which are often relied upon to support reproducibility across laboratories [6,17]. The commercial electrode used in this study was selected as a comparator based on availability within established workflows and should not be interpreted as a universal standard across laboratories.

### 3.6. Future Directions

Future work could include multi-site validation, testing in additional muscle groups, and systematic exploration of stimulation parameters. For the StimJim, the evaluation of alternative pulse widths, waveforms, and combining two outputs in series may help narrow twitch differences. Formal durability testing of reusable electrodes would also be useful. Furthermore, since some laboratories use nerve-targeted subcutaneous/percutaneous needle or wire electrodes for NMES of the hindlimb ankle dorsiflexors [18,19], it would be worth comparing those methods with muscle-belly-targeted stimulation with the StimJim.

## 4. Materials and Methods

### 4.1. Research Design

This project consisted of two complementary studies designed to evaluate accessible tools for transcutaneous neuromuscular electrical stimulation (NMES) in mice. The first study compared three bipolar electrode configurations for murine NMES (electrode study). The second study compared two electrical stimulators and included development of a practical workflow for murine NMES using an open-source stimulator (stimulator study). Both studies focused on measurement of contractile torque of the ankle dorsiflexor muscle group, for which the tibialis anterior (TA) is the primary contributor. Contractile torque measurements were performed with a custom-built dynamometry device that has been described previously [4,5,6].

These studies were designed as methodological validation experiments rather than mechanistic or preclinical studies. Sample sizes were therefore consistent with prior reproducibility and device-validation studies in murine NMES, where within-animal repeated-measures designs improved statistical efficiency and reduced animal numbers. Experimental workflows were predefined to promote consistency across animals and to minimize operator-dependent variability.

### 4.2. Animal Models

All animal procedures were conducted at Wayne State University (Detroit, MI, USA) under protocols approved by the Institutional Animal Care and Use Committee (IACUC). All procedures conformed to the Guide for the Care and Use of Laboratory Animals (8th Edition, National Academies Press, 2011).

Only male mice were studied. This decision was based on prior literature demonstrating that responses of healthy and dystrophic mice to electrically induced muscle contractions, including eccentric contractions, are well characterized in male animals [20]. Sex-related differences in skeletal muscle response and injury susceptibility have been reported, with female mice demonstrating altered responses that may introduce additional variability [21,22,23,24]. Using male mice also facilitated comparison with previously published NMES datasets from our laboratory and others [20].

### 4.3. Electrode Study

Adult male C57BL/6J mice (*N* = 4; Stock No. 000664, The Jackson Laboratory, Bar Harbor, ME, USA) and dysferlin-null B6.A-Dysf^prmd^/GeneJ (BLAJ) mice (*N* = 4; Stock No. 012767, The Jackson Laboratory) were studied. BLAJ mice were provided as a gift by the Jain Foundation Inc., which maintains a colony of these animals at The Jackson Laboratory.

C57BL/6J mice served as healthy controls. BLAJ mice served as a disease-relevant comparison strain modeling dysferlin-related muscular dystrophy. BLAJ mice carry the A/J dysferlin mutation on a C57BL/6J background and are syngeneic, making them suitable for comparisons with C57BL/6J mice while representing a clinically relevant dystrophic phenotype [9,10].

Mice were 3–4 months of age at the time of testing. At this age, mice have a mature musculoskeletal system while exhibiting minimal age-related decline in contractile function. For dystrophic models such as BLAJ mice, this age also precedes substantial spontaneous muscle pathology, thereby potentially reducing the confounding effects of ongoing degeneration [9].

### 4.4. Stimulator Study

A convenience sample of adult male C57BL/6J mice (*N* = 8; approximately 1 year old; Stock No. 000664, The Jackson Laboratory) was studied. Older animals were acceptable for this study because the primary goal was device comparison rather than genotype comparison. These animals differed in age from those used in the electrode study; however, comparisons were performed within-animal for each device, minimizing confounding.

### 4.5. Animal Anesthesia, Preparation, and Positioning

Detailed methodological descriptions (text and audiovisual material) on how animals are prepared for NMES and torque measurement have been published earlier [5]. All procedures were performed under inhaled isoflurane anesthesia (tabletop isoflurane vaporizer, VetEquip, Livermore, CA, USA). Anesthesia was induced using 2–5% isoflurane and maintained at 1–4% via a nose cone. Oxygen flow was maintained at approximately 1–2 L/min, and a carbon canister scavenging system was used to reduce rebreathing of isoflurane. Depth of anesthesia was assessed by toe pinch and visual assessment of respiratory rate and depth. Thermal support was provided with an isothermal placed beneath the animal, and a heat lamp placed ~1 m above the animal. A thermometer placed on the acrylic base of the testing rig was used to periodically confirm that the temperature around the animal was ~38 °C. Animals were allowed to recover individually with thermal support (Deltaphase isothermal pad, Braintree Scientific, Braintree, MA, USA) in a bedding-free cage and returned to their original cage after regaining consciousness and mobility.

### 4.6. Contractile Torque Measurement

Torque produced by the ankle dorsiflexors was measured using a custom-built robotic dynamometer [5,6]. The system consisted of a rigid footplate connected to a torque cell and stepper motor. Methodological details on the dynamometry device and relevant techniques have been documented in prior publications [4,5,6] and illustrated with a video in Supplementary Materials.

Custom-written software in LabVIEW 2014 (National Instruments, Austin, TX, USA) was used to maintain ankle position, trigger stimulation, and record torque output in real time. Torque signals were visualized during testing to confirm consistent contractions and stored digitally for later analysis.

For peroneal nerve stimulation, electrodes were placed over the lateral hindlimb distal to the knee. A palpable bony prominence served as a positioning landmark. Correct placement was verified by delivering 1 Hz stimulation and observing visible dorsiflexion twitches of the foot and corresponding torque readings, indicating activation of the dorsiflexor muscle group. This standard procedure had to be altered while using the StimJim—the electrode had to be placed directly over the TA muscle belly.

### 4.7. Electrical Stimulation

#### 4.7.1. Determination of Optimal Intensity

For each animal and condition, stimulation amplitude was gradually increased while monitoring twitch torque. Current or voltage was increased until torque reached a plateau, indicating maximal motor unit recruitment. The lowest intensity that produced a stable maximal twitch torque was used for subsequent trials.

#### 4.7.2. Grass S48 Stimulator

Square pulses were generated using a Grass S48 stimulator coupled to a PSIU6 stimulation isolation unit. This system has been used in many small-animal NMES studies and served as the reference stimulator [5,6,25,26].

Pulse duration was 0.1 ms (100 µs). Twitch contractions were elicited at 1 Hz. Tetanic contractions were elicited at 125 Hz with a 450 ms train duration. Stimulation intensity was increased until maximal torque was achieved. The Grass S48 system was used exclusively in the electrode study and served as the comparator in the stimulator study.

#### 4.7.3. StimJim Open-Source Stimulator

NMES was also performed using the open-source StimJim stimulator, controlled by a Teensy 3.5 microcontroller and programmed via USB connection to a host computer.

Simplified firmware derived from the StimJim pulser example firmware was used to enable reliable parameter entry. Coding files and the steps involved in starting up the StimJim, installing firmware, and command structure to enter stimulation parameters are provided as Supplementary Materials (File S1).

Stimulation was delivered in monophasic square pulses (to match Grass S48) in constant-current mode. Initial attempts were made to match the 0.1 ms pulse duration used with the Grass S48. Pilot testing indicated that longer pulse durations were required to achieve optimal stimulation that generated stable torque readings. A pulse width of 1 ms (1000 µs) was therefore used for all StimJim experiments—this parameter difference reflects workflow optimization for each device rather than an attempt to match pulse duration directly. The StimJim can be used either in constant voltage or constant current mode. During exploratory testing, we tried using the StimJim in constant voltage mode to match the Grass S48, but switched to constant current mode since we found torque readings to be more stable.

Twitch contractions were elicited at 1 Hz and tetanic contractions at 125 Hz with a 450 ms train duration. Current amplitude was increased individually until maximal twitch torque was obtained, typically in the range of 2–3 mA.

For StimJim trials, electrodes were positioned directly over the tibialis anterior muscle belly. This placement provided more consistent recruitment than nerve-targeted placement while using the StimJim.

Investigators were not blinded to stimulator type due to obvious differences in hardware appearance and operation.

### 4.8. Electrode Designs

#### 4.8.1. Commercial Electrode

A previously available bipolar electrode (Simple Electrode, BS4 50–6824, Harvard Apparatus) served as the commercial reference comparator in this study. This electrode is no longer commercially available [5].

#### 4.8.2. 3D-Printed Electrode

A custom bipolar electrode, based on the design of the discontinued Simple Electrode, was designed using Tinkercad (Autodesk, Inc., San Rafael, CA, USA, https://www.tinkercad.com), and exported as an STL file. Files were processed using Ultimaker Cura to generate printer-specific G-code (Ultimaker B.V., Utrecht, The Netherlands). Mirror-image halves were printed in PLA filament (HATCHBOX 1.75 mm Silver PLA Filament, HATCHBOX, distributed via Amazon/3D Printing Canada, Rowland Heights, CA, USA) on a delta-style 3D printer (FLSUN SR Delta 3D Printer; FLSUN, Shanghai, China). Each half of the 3D-printed electrode housing had grooves designed to hold 20-gauge stainless steel wire terminals. Wire segments were manually straightened with plastic-coated jewelry pliers and deburred using a rotary sanding disc to remove sharp edges. Wires were placed in the grooves, and the halves were bonded with cyanoacrylate adhesive to form a rigid insulated housing with fixed inter-electrode spacing. Design files are provided as Supplementary Materials under a CC BY-NC 3.0 license. A video demonstration of the use of the 3D-Printed electrode in NMES is also included under Supplementary Materials.

#### 4.8.3. Pen Electrode

The Pen electrode was assembled from paired male header pins and jumper wires housed within a plastic pen barrel. Jumper wires were threaded through the barrel and stabilized with hot glue. The exposed header prongs served as skin-contact terminals. This design was developed as an ultra-low-cost and rapidly assembled option using commonly available laboratory components.

### 4.9. Electrode Wiring

Electrodes were connected to the stimulator isolation unit using approximately one meter of 26 AWG two-conductor wire. Jumper connectors enabled rapid electrode exchange. Heat-shrink tubing was used to insulate and color-code connections to reduce wiring errors.

### 4.10. Experimental Workflows

For the electrode study, each animal underwent testing with all three electrode types. Electrode order was randomized within animals using a simple randomized sequence. After confirming correct placement with 1 Hz stimulation, twitch contractions were elicited first, followed by tetanic contractions. Rest intervals of at least three minutes were provided between trials to minimize fatigue. Multiple contractions (typically three) were recorded for each condition, and the highest reproducible torque value (best of three stable readings) was selected for analysis.

For the stimulator study, each animal was tested with both stimulators under matched conditions in random order. Optimal stimulation intensity was determined individually for each device. Twitch contractions were elicited first, followed by tetanic contractions. Rest intervals of at least three minutes were provided between stimulator conditions. Peak torque values were recorded for analysis.

Investigators were not blinded to electrode or stimulator type due to visible differences in device construction.

### 4.11. Statistical Analysis

Torque data were compiled in Microsoft Excel (Microsoft Corporation, Redmond, WA, USA) and analyzed using SigmaStat 3.5 (Systat Software, Inc., San Jose, CA, USA). For the electrode study, we performed two-way repeated-measures ANOVA with factors of electrode type and genotype. Holm–Sidak post hoc tests were applied when appropriate. For the stimulator study, we performed paired comparisons. Normality was assessed prior to testing. When assumptions were met, paired *t*-tests were used. When normality assumptions failed, Wilcoxon signed-rank tests were applied. We defined statistical significance as *p* < 0.05, a priori.

### 4.12. Artificial Intelligence (AI) Use

AI-assisted tools (ChatGPT, OpenAI; version 5.2) were used for language editing and code refinement. Figure 1A was created with BioRender.com and embedded AI. All scientific content, methods, code, and conclusions were developed, verified, and approved by the authors. The authors take full responsibility for all content.

## 5. Conclusions

This study provides practical alternatives for two key components of murine NMES—bipolar transcutaneous electrodes and a laboratory stimulator. Both custom electrodes that we designed and tested produced tetanic torque similar to a discontinued commercial electrode in healthy and dysferlin-deficient mice, supporting their use when maximal force is the primary outcome. The open-source StimJim produced similar tetanic torque to the discontinued Grass S48 under the tested conditions, although twitch torque was lower and a defined workflow (e.g., electrode over muscle belly and higher pulse duration) was required to achieve optimal stimulation and stable torque readings.

By providing construction details and practical stimulation workflows, this work may help laboratories pursue murine NMES research despite equipment discontinuation and/or funding limitations. Additional validation across laboratories and experimental contexts will further define the generalizability of these approaches.

## Data Availability

All relevant supporting data have been provided as Supplementary Materials.

## Supplementary Materials

The following supporting information can be downloaded at: https://www.mdpi.com/article/10.3390/muscles5020032/s1, Supplementary Materials have been submitted by the authors and will be hosted by the journal. Design files: 3D-printing-related information for bipolar electrode. Video file: NMES with 3D-Printed electrode. Coding files: Pulser Arduino code for constant current and constant voltage modes in StimJim. Supplementary methods: File S1, setup and operation of the StimJim for murine NMES. Figure S1: 3D-printed electrode design; Table S1: Calculation of Effect Sizes for Paired Differences (Cohen’s dz) in the Electrode Study; Table S2: Calculation of Effect Sizes for Paired Differences (Cohen’s dz) in the Stimulator Study. Video S1: Demonstration of NMES on the hindlimb ankle dorsiflexors in a mouse.

## Abbreviations

The following abbreviations are used in this manuscript:

NMES	Neuromuscular electrical stimulation
TA	Tibialis anterior
IACUC	Institutional Animal Care and Use Committee
