# Supplementary material for: Open-Source Tools for Neuromuscular Electrical Stimulation in Mouse Models: A Methodological Validation Study
Source: Muscles. 2026 Apr 30;5(2):32. doi: 10.3390/muscles5020032 (PMC13214815; doi:10.3390/muscles5020032)
Supplement: Supplementary file 1 [file muscles-05-00032-s001.zip › Figure S1_Electrode Design.pdf]

TINKER CAD LINK:

https://www.tinkercad.com/things/9EEQGKuZWgi-roche-lab-trapezoidal-bipolar-electrode-v-121224?sharecode=wJ7C56aSKXSdAVgP6K111cjZVsC_w3x1GuVSCGdHaKI

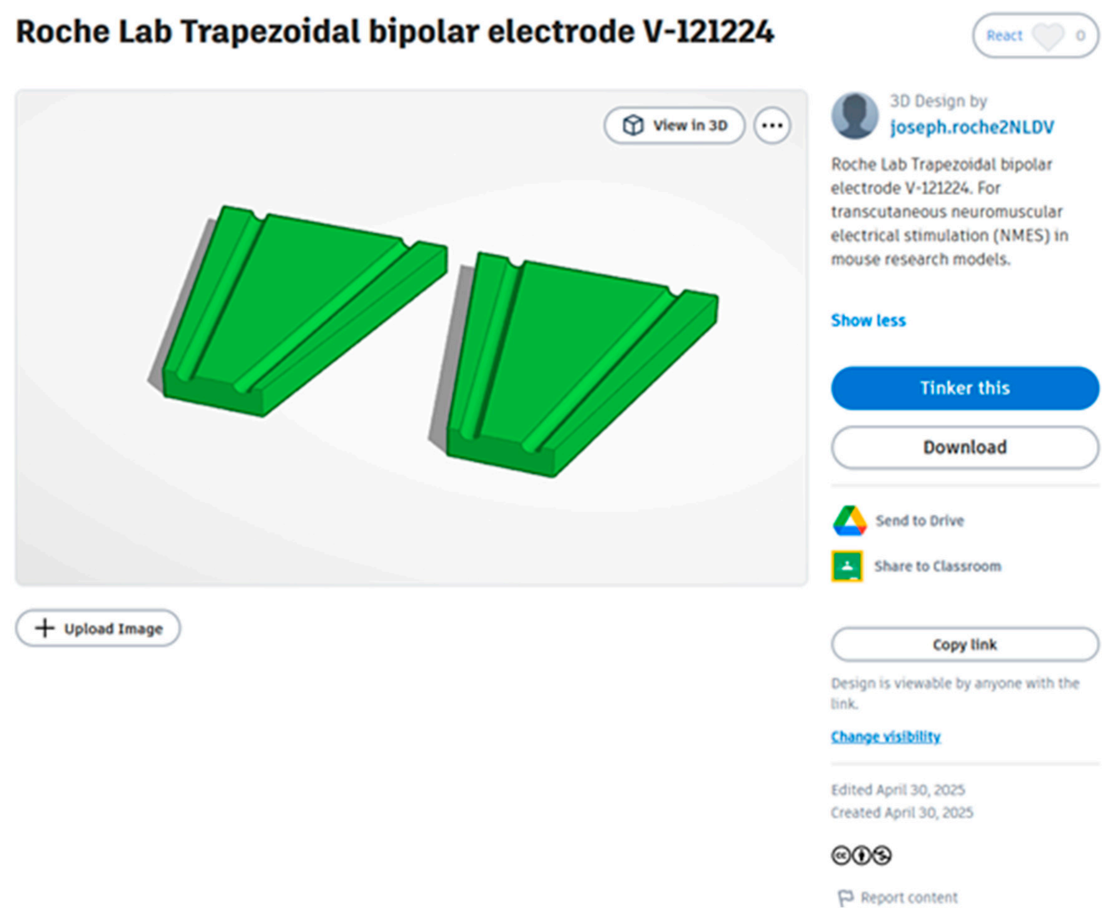


License: https://creativecommons.org/licenses/by-nc/3.0/
