# Supplementary material for: Open-Source Tools for Neuromuscular Electrical Stimulation in Mouse Models: A Methodological Validation Study
Source: Muscles. 2026 Apr 30;5(2):32. doi: 10.3390/muscles5020032 (PMC13214815; doi:10.3390/muscles5020032)
Supplement: Supplementary file 1 [file muscles-05-00032-s001.zip › S1_SUPPL METHODS.pdf]

Supplementary Methods S1

Setup and operation of the StimJim for murine NMES

S1.1. Purpose and Scope

This supplementary guide provides a workflow for configuring and operating the StimJim open-source stimulator for murine neuromuscular electrical stimulation (NMES). The goal is to facilitate adoption of the StimJim platform in NMES and related applications. Hardware connections, electrode placement, and dynamometry setup are described in the main manuscript. This guide focuses on software setup, device recognition, and stimulation parameter entry.

S1.2. Required Hardware

- StimJim stimulator (Open Ephys)
- Teensy microcontroller (installed in StimJim)
- Micro-USB cable (data capable)
- Host computer (Windows-based system used in this study; other operating systems may be compatible but were not tested)
- Bipolar stimulation electrode, such as our 3D-Printed electrode
- Connection leads between stimulator and electrode

S1.3. Required Software

- Arduino integrated development environment (IDE)
- StimJim pulser firmware (inventor-provided pulser or modified version used in this study; serial command structures may differ between firmware versions)
- USB drivers for Teensy

S1.4. Connecting the StimJim (images in main paper, Fig.2)

1. Connect the StimJim to the host computer using a micro-USB cable.
2. Confirm that the device powers on.
3. Allow the operating system to recognize the Teensy microcontroller.

S1.5. Launching the Arduino IDE

1. Open the Arduino IDE.

2. Load the StimJim pulser firmware (.ino file, either constant current or voltage based on requirement. We used constant current for data collection).Confirm that the code compiles without errors.

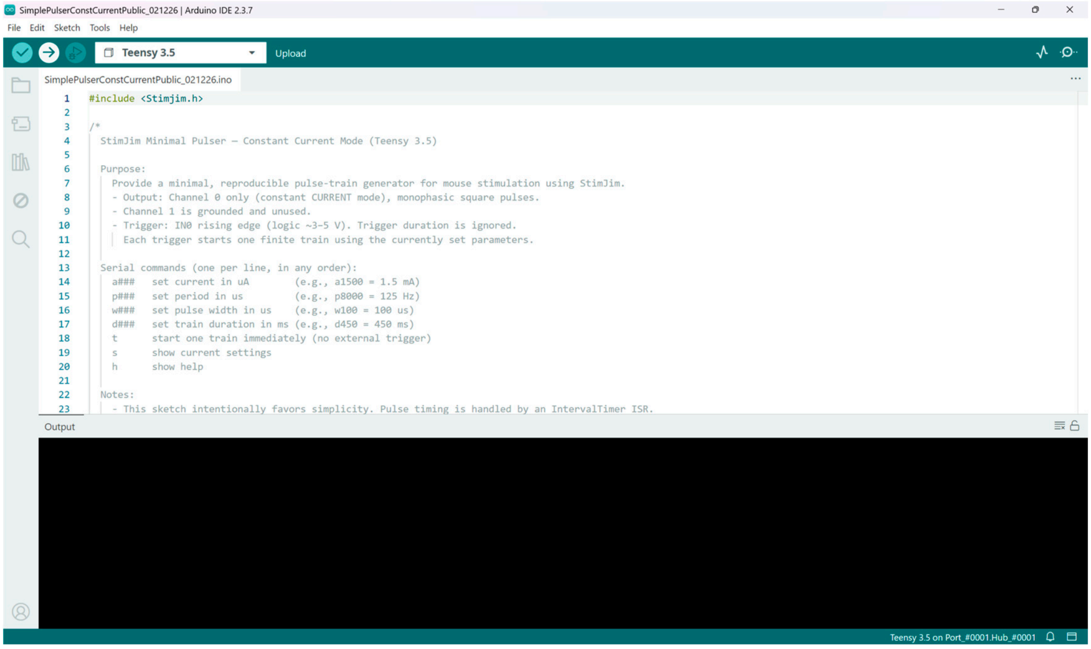

S1.6. Selecting the Board

1. In the Arduino IDE, navigate to:

   Tools → Board

2. Select the appropriate Teensy board model (e.g., Teensy 3.5 or actual model used in StimJim).

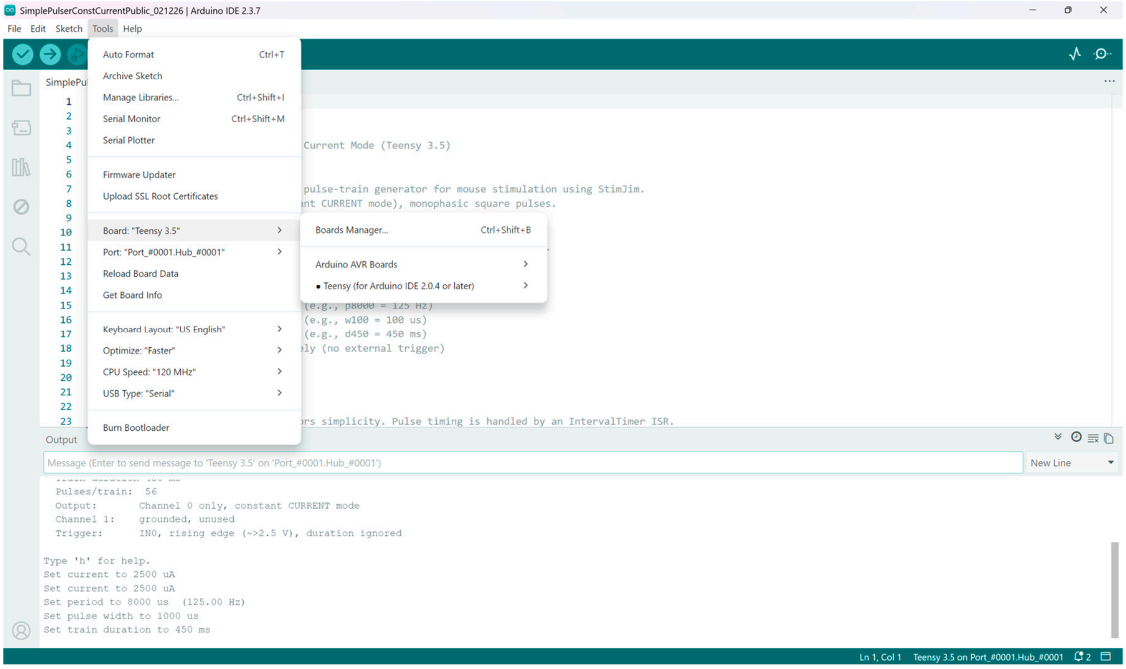

S1.7. Selecting the Port

1. Navigate to:

   Tools → Port

2. Select the port associated with the StimJim/Teensy.

3. If multiple ports appear, unplug and reconnect the device to identify the correct one.

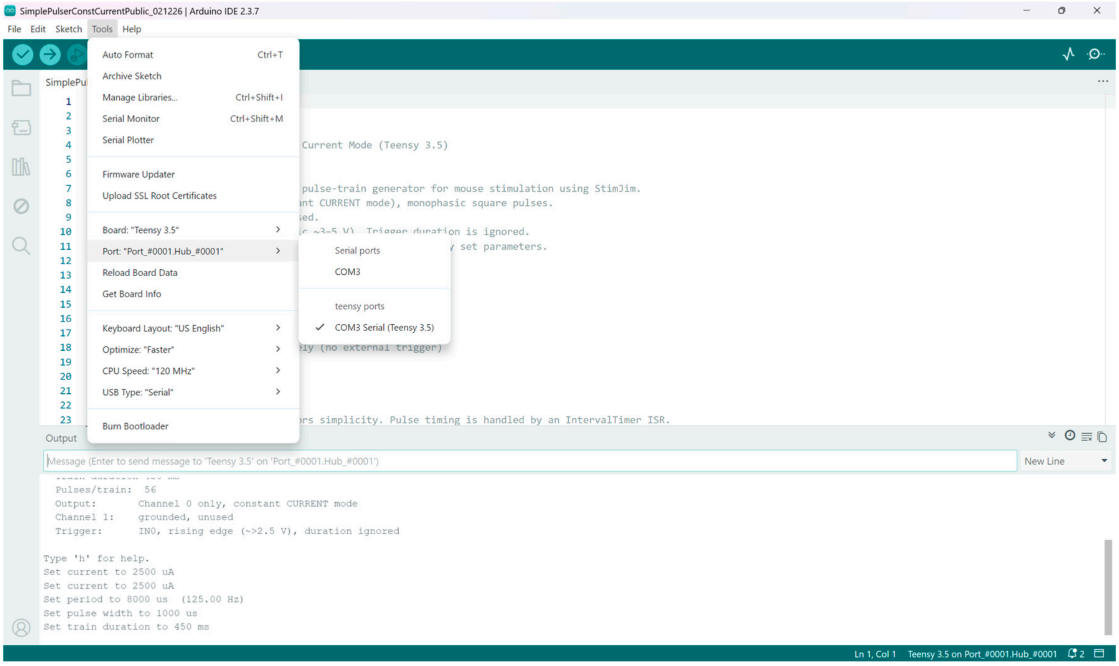

S1.8. Uploading Firmware

1. Click Upload in the Arduino IDE.

2. Wait for confirmation that upload is complete.

3. If upload fails, recheck board and port selections.

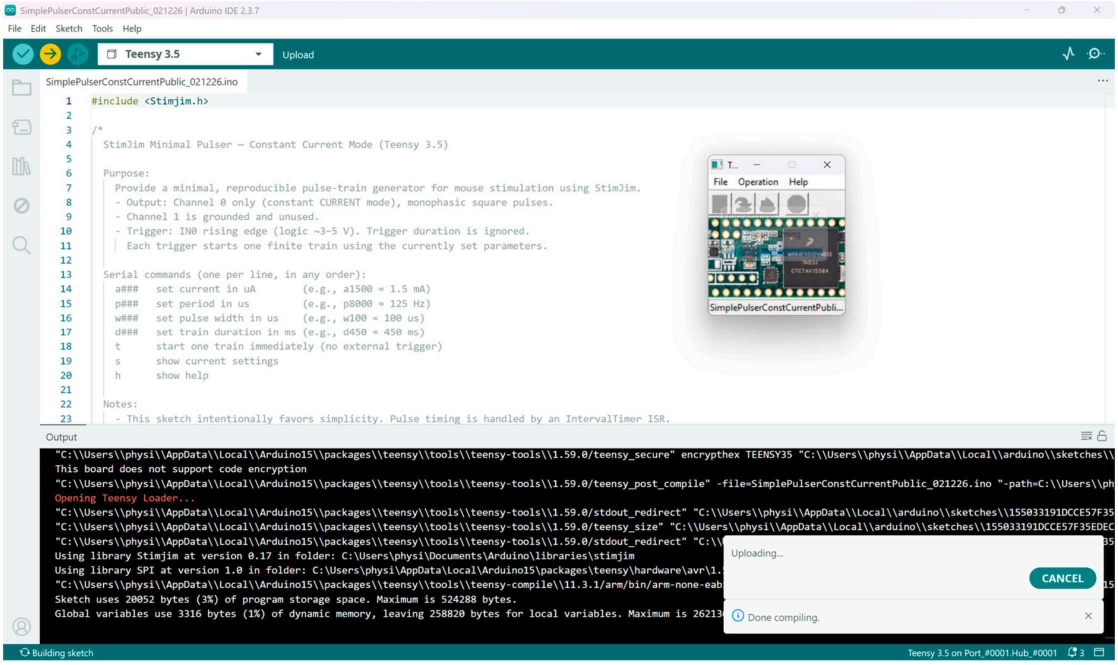

S1.9. Opening the Serial Monitor

1. Open the Serial Monitor in Arduino IDE.

2. Confirm that the baud rate matches the firmware settings.

3. Verify that the StimJim responds to serial input.

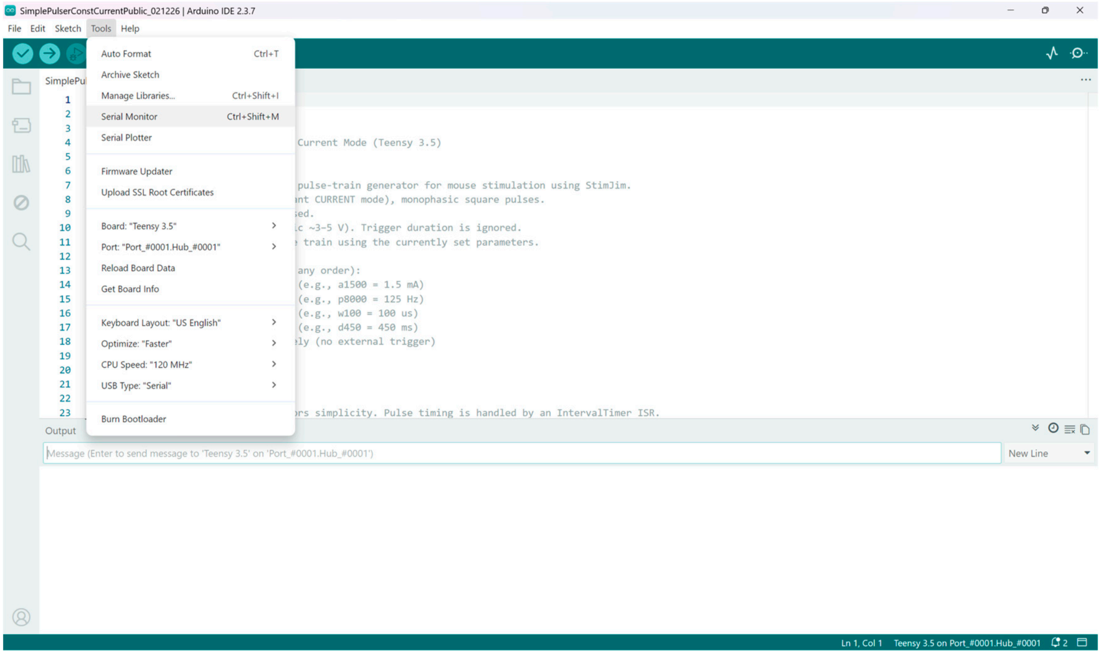

S1.10. Entering Stimulation Parameters

Stimulation parameters are defined via serial commands. These include:

- Pulse width
- Current or voltage amplitude
- Frequency
- Train duration

In the pulsers that we have provided, commands are entered as plain text and sent via the Serial Monitor, one parameter at a time. Details are provided, below, under specific Constant Current and Voltage Pulsers.

S1.10.1. Constant-Current Mode

1. Enter command structure for constant-current stimulation in Serial Monitor window. Enter one parameter at a time, hitting enter after each parameter. e.g., for tetanic stimulation at 125Hz frequency, enter: a1500 for current amplitude of 1.5mA; p8000 for period of 8000µs or frequency of 125Hz (frequency = 1,000,000/period); w1000 for pulse width or duration of 1000µs or 1ms; and d450 for pulse train duration of 450ms.

2. Confirm that the device acknowledges the command.

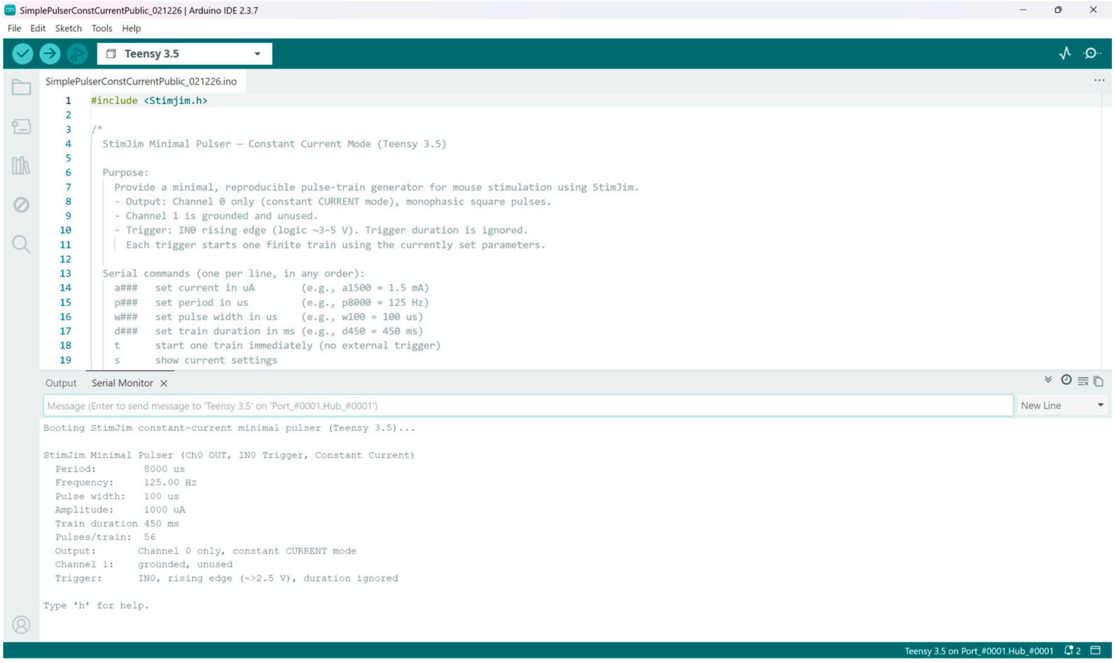

S1.10.2. Constant-Voltage Mode

1. Enter command structure for constant-voltage stimulation in Serial Monitor window. Enter one parameter at a time, hitting enter after each parameter. e.g., for tetanic stimulation at 125Hz frequency, enter: v10000 for voltage amplitude of 10V; p8000 for period of 8000µs or frequency of 125Hz (frequency = 1,000,000/period); w1000 for pulse width or duration of 1000µs or 1ms; and d450 for pulse train duration of 450ms.
2. Confirm device response.

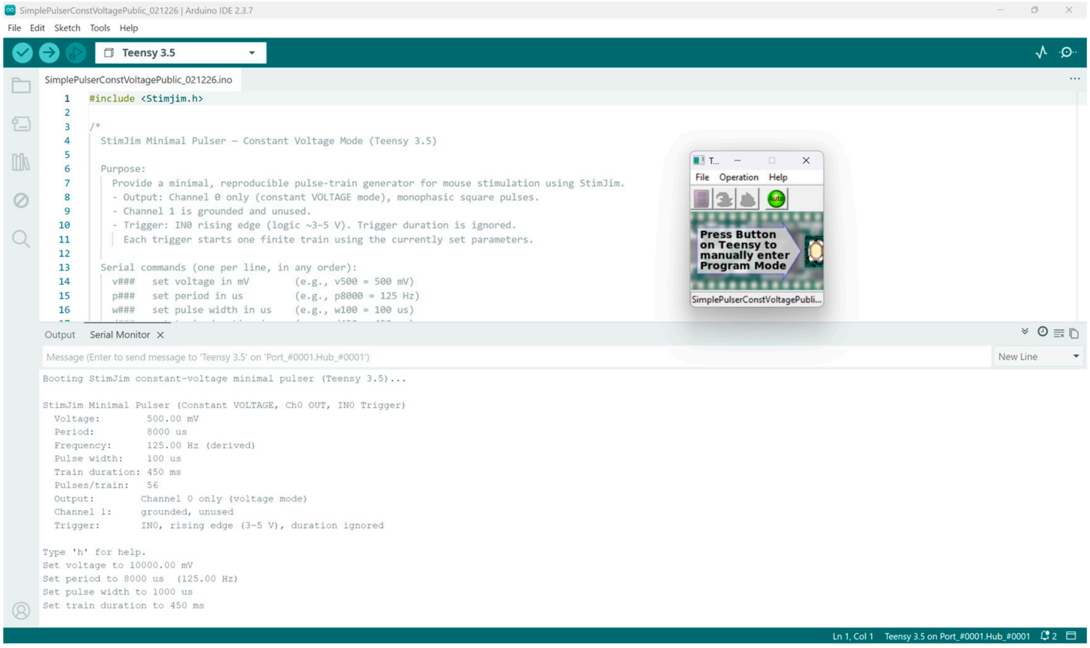

S1.11. Verifying output

1. Connect the stimulator output to the electrode.
2. Deliver a test pulse.
3. Confirm muscle contraction or oscilloscope output if available.
4. Adjust parameters as needed.

S1.12. Practical Notes for First-Time Users

- In our setup, the pulser output was assigned to Channel 0. Input 0 was used to receive a 3–5 V trigger signal from the dynamometry system. This allowed stimulation to occur only when triggered. Laboratories without a robotic dynamometry system may use a 4.5 V battery pack and a manual or automated switch as an alternative trigger source.
- Confirm board and port selection before uploading firmware.
- Use a data-capable USB cable.
- Stable recruitment may require longer pulse widths than legacy stimulators.
- If torque drops abruptly, reassess electrode placement (e.g., try placing electrode over muscle belly as described in our paper) and cable connections.

S1.13. Basic Troubleshooting

Issue	Possible Cause	Suggested Action
Device not recognized	USB cable or driver issue	Try a different cable/port
Firmware upload fails	Wrong board/port	Re-select board and port
No stimulation output	Parameter error	Re-enter commands
Unstable contractions	Electrode contact	Reposition electrodes
Unstable contractions	Low pulse width/duration (e.g., incorrectly set to 100μs instead of 1000μs)	Change pulse width/duration to 1000μs
Unstable contractions	Amplitude not optimal	Optimize current or voltage amplitude such that maximal twitch torque is obtained with the lowest amplitude needed.

S1.14. Connection to Main Methods

This workflow reflects the practical setup used in the present study. Detailed stimulation parameters and experimental protocols are described in the main Methods section.
