# Supplementary material for: Open-Source Tools for Neuromuscular Electrical Stimulation in Mouse Models: A Methodological Validation Study
Source: Muscles. 2026 Apr 30;5(2):32. doi: 10.3390/muscles5020032 (PMC13214815; doi:10.3390/muscles5020032)
Supplement: Supplementary file 1 [file muscles-05-00032-s001.zip › Suppl Table S1_Electrode Study.pdf]

Supplementary Table S1. Electrode Study. Calculation of Effect Sizes for Paired Differences (Cohen's dz).

**TWITCH C57BL/6J**							
**ANIMAL ID**	**Commercial**	**3D-Printed**	**Pen**		**ANIMAL ID**	**Commercial Minus 3D-Printed**	**Commercial Minus Pen**
C57 01	763	769	549		C57 01	-6	214
C57 02	669	652	672		C57 02	17	-3
C57 03	541	627	567		C57 03	-86	-26
C57 04	696	617	502		C57 04	80	194
MEAN	668	666	573		MEAN	1	95
STD. DEV.	93	70	72		STD. DEV.	68	127
COUNT	4	4	4		COUNT	4	4
CONFIDENCE.T	148	112	114		CONFIDENCE.T	109	202
CI UPPER BOUND	816	778	687		CI UPPER BOUND	110	297
CI LOWER BOUND	520	555	458		CI LOWER BOUND	-107	-107
					**Paired Cohen's d (dz)**	**0.02**	**0.75**
**TWITCH BLAJ**							
**ANIMAL ID**	**Commercial**	**3D-Printed**	**Pen**		**ANIMAL ID**	**Commercial Minus 3D-Printed**	**Commercial Minus Pen**
BLAJ 01	713	698	570		BLAJ 01	15	143
BLAJ 02	649	645	579		BLAJ 02	4	70
BLAJ 03	642	688	595		BLAJ 03	-46	46
BLAJ 04	374	343	385		BLAJ 04	31	-11
MEAN	594	594	532		MEAN	1	62
STD. DEV.	150	168	99		STD. DEV.	33	64
COUNT	4	4	4		COUNT	4	4
CONFIDENCE.T	239	268	157		CONFIDENCE.T	53	101
CI UPPER BOUND	833	862	690		CI UPPER BOUND	54	163
CI LOWER BOUND	356	326	375		CI LOWER BOUND	-52	-39
					**Paired Cohen's d (dz)**	**0.02**	**0.98**

Supplementary Table S1. Electrode Study. Calculation of Effect Sizes for Paired Differences (Cohen's dz).

**TETANY C57BL/6J**							
**ANIMAL ID**	**Commercial**	**3D-Printed**	**Pen**		**ANIMAL ID**	**Commercial Minus 3D-Printed**	**Commercial Minus Pen**
C57 01	2454	2450	2420		C57 01	4	34
C57 02	1814	1923	1876		C57 02	-109	-62
C57 03	2046	2006	2039		C57 03	40	7
C57 04	1915	1931	1710		C57 04	-16	205
MEAN	2057	2077	2011		MEAN	-20	46
STD. DEV.	281	251	304		STD. DEV.	64	114
COUNT	4	4	4		COUNT	4	4
CONFIDENCE.T	447	399	483		CONFIDENCE.T	101	181
CI UPPER BOUND	2504	2477	2495		CI UPPER BOUND	81	226
CI LOWER BOUND	1610	1678	1528		CI LOWER BOUND	-122	-135
					**Paired Cohen's d (dz)**	**-0.32**	**0.40**
**TETANY BLAJ**							
**ANIMAL ID**	**Commercial**	**3D-Printed**	**Pen**		**ANIMAL ID**	**Commercial Minus 3D-Printed**	**Commercial Minus Pen**
BLAJ 01	2859	2772	2716		BLAJ 01	86	142
BLAJ 02	2540	2521	2127		BLAJ 02	19	412
BLAJ 03	2647	2491	2621		BLAJ 03	156	26
BLAJ 04	2223	2203	2025		BLAJ 04	20	198
MEAN	2567	2497	2373		MEAN	70	195
STD. DEV.	265	233	347		STD. DEV.	65	162
COUNT	4	4	4		COUNT	4	4
CONFIDENCE.T	421	370	552		CONFIDENCE.T	104	258
CI UPPER BOUND	2989	2867	2924		CI UPPER BOUND	174	452
CI LOWER BOUND	2146	2126	1821		CI LOWER BOUND	-34	-63
					**Paired Cohen's d (dz)**	**1.08**	**1.20**
