# Supplementary material for: Open-Source Tools for Neuromuscular Electrical Stimulation in Mouse Models: A Methodological Validation Study
Source: Muscles. 2026 Apr 30;5(2):32. doi: 10.3390/muscles5020032 (PMC13214815; doi:10.3390/muscles5020032)
Supplement: Supplementary file 1 [file muscles-05-00032-s001.zip › Suppl Table S2_Stimulator Study.pdf]

Supplementary Table S2. Stimulator Study. Calculation of Effect Sizes for Paired Differences (Cohen's dz).

**TWITCH**					
**Animal ID**	**Grass S48**	**StimJim**		**Animal ID**	**Grass S48 Minus StimJim**
Animal 1	716	612		Animal 1	104
Animal 2	943	785		Animal 2	158
Animal 3	584	468		Animal 3	116
Animal 4	586	577		Animal 4	9
Animal 5	737	686		Animal 5	51
Animal 6	712	563		Animal 6	149
Animal 7	691	537		Animal 7	154
Animal 8	618	473		Animal 8	144
AVERAGE	698	588		AVERAGE	111
STD.DEV	116	107		STD.DEV	55
COUNT	8	8		COUNT	8
CONFIDENCE.T	97	89		CONFIDENCE.T	46
CI UPPER BOUND	795	677		CI UPPER BOUND	156
CI LOWER BOUND	601	498		CI LOWER BOUND	65
				**Paired Cohen's d (dz)**	**2.03**

Supplementary Table S2. Stimulator Study. Calculation of Effect Sizes for Paired Differences (Cohen's dz).

**TETANY**					
**Animal ID**	**Grass S48**	**StimJim**		**Animal ID**	**Grass S48 Minus StimJim**
Animal 1	2375	2274		Animal 1	101
Animal 2	3270	3173		Animal 2	98
Animal 3	2845	2851		Animal 3	-6
Animal 4	3267	2997		Animal 4	270
Animal 5	2611	2581		Animal 5	29
Animal 6	3022	2863		Animal 6	159
Animal 7	2571	1661		Animal 7	910
Animal 8	2201	2253		Animal 8	-52
AVERAGE	2770	2581		AVERAGE	189
STD.DEV	398	496		STD.DEV	308
COUNT	8	8		COUNT	8
CONFIDENCE.T	333	415		CONFIDENCE.T	258
CI UPPER BOUND	3103	2996		CI UPPER BOUND	447
CI LOWER BOUND	2437	2167		CI LOWER BOUND	-69
				**Paired Cohen's d (dz)**	**0.61**
